# Supplementary material for: Anionic phospholipids control mechanisms of GPCR-G protein recognition
Source: Nat Commun. 2023 Feb 13;14:794. doi: 10.1038/s41467-023-36425-z (PMC9925817; doi:10.1038/s41467-023-36425-z)
Supplement: Supplementary file 3 — Reporting Summary [file 41467_2023_36425_MOESM3_ESM.pdf]

nature portfolio

Corresponding author(s): Matthew Eddy

Last updated by author(s): Jan 19, 2023

Reporting Summary

Nature Portfolio wishes to improve the reproducibility of the work that we publish. This form provides structure for consistency and transparency in reporting. For further information on Nature Portfolio policies, see our Editorial Policies and the Editorial Policy Checklist.

Statistics

For all statistical analyses, confirm that the following items are present in the figure legend, table legend, main text, or Methods section.

n/a	Confirmed	
☐	☒	The exact sample size (*n*) for each experimental group/condition, given as a discrete number and unit of measurement
☐	☒	A statement on whether measurements were taken from distinct samples or whether the same sample was measured repeatedly
☒	☐	The statistical test(s) used AND whether they are one- or two-sided<br>*Only common tests should be described solely by name; describe more complex techniques in the Methods section.*
☒	☐	A description of all covariates tested
☒	☐	A description of any assumptions or corrections, such as tests of normality and adjustment for multiple comparisons
☐	☒	A full description of the statistical parameters including central tendency (e.g. means) or other basic estimates (e.g. regression coefficient) AND variation (e.g. standard deviation) or associated estimates of uncertainty (e.g. confidence intervals)
☒	☐	For null hypothesis testing, the test statistic (e.g. *F*, *t*, *r*) with confidence intervals, effect sizes, degrees of freedom and *P* value noted<br>*Give P values as exact values whenever suitable.*
☒	☐	For Bayesian analysis, information on the choice of priors and Markov chain Monte Carlo settings
☒	☐	For hierarchical and complex designs, identification of the appropriate level for tests and full reporting of outcomes
☒	☐	Estimates of effect sizes (e.g. Cohen's *d*, Pearson's *r*), indicating how they were calculated

Our web collection on statistics for biologists contains articles on many of the points above.

Software and code

Policy information about availability of computer code

Data collection	For NMR data collection, Bruker Topspin 3.6.2 was used. Molecular simulation data were generated with Gromacs 2020.4; lipids and protein were modeled with the CHARMM36 force field. No new codes were generated for this study.
Data analysis	For NMR data processing and analysis, Bruker Topspin 3.6.2 and MestreNova version 14.1.1-24571 were used. Pharmacological data and cAMP data were processed and analyzed in GraphPad Prism version 8. Molecular simulation data were analyzed with CHARMMGUI. Thermal shift assay data were analyzed in Origin 2022.

For manuscripts utilizing custom algorithms or software that are central to the research but not yet described in published literature, software must be made available to editors and reviewers. We strongly encourage code deposition in a community repository (e.g. GitHub). See the Nature Portfolio guidelines for submitting code & software for further information.

Data

Policy information about availability of data

All manuscripts must include a data availability statement. This statement should provide the following information, where applicable:

- Accession codes, unique identifiers, or web links for publicly available datasets
- A description of any restrictions on data availability
- For clinical datasets or third party data, please ensure that the statement adheres to our policy

Source data are provided in the accompanying file. NMR and molecular dynamics source data are made available through the Open Science Framework data repository. Information on how to access the files, including a permanant link to the files, is provided in the main text in the data availability section.

Human research participants

Policy information about studies involving human research participants and Sex and Gender in Research.

Reporting on sex and gender	N/A
Population characteristics	N/A
Recruitment	N/A
Ethics oversight	N/A

Note that full information on the approval of the study protocol must also be provided in the manuscript.

Field-specific reporting

Please select the one below that is the best fit for your research. If you are not sure, read the appropriate sections before making your selection.

☒ Life sciences ☐ Behavioural & social sciences ☐ Ecological, evolutionary & environmental sciences

For a reference copy of the document with all sections, see nature.com/documents/nr-reporting-summary-flat.pdf

Life sciences study design

All studies must disclose on these points even when the disclosure is negative.

Sample size	No sample-size calculations were performed. Sample size was determined to be adequate based on the magnitude and consistency of measurable differences between groups.
Data exclusions	No data were excluded from the study.
Replication	Experiments utilizing statistical analysis were done in triplicate and were reproducible.
Randomization	Experiments were planned systematically with appropriate controls. No randomization was involved.
Blinding	The data and inferences in this study were analyzed and generated objectively. Hence blinding is not applicable as there is no bias present.

Reporting for specific materials, systems and methods

We require information from authors about some types of materials, experimental systems and methods used in many studies. Here, indicate whether each material, system or method listed is relevant to your study. If you are not sure if a list item applies to your research, read the appropriate section before selecting a response.

Materials & experimental systems

n/a	Involved in the study
☒	☐ Antibodies
☐	☒ Eukaryotic cell lines
☒	☐ Palaeontology and archaeology
☒	☐ Animals and other organisms
☒	☐ Clinical data
☒	☐ Dual use research of concern

Methods

n/a	Involved in the study
☒	☐ ChIP-seq
☒	☐ Flow cytometry
☒	☐ MRI-based neuroimaging

Eukaryotic cell lines

Policy information about cell lines and Sex and Gender in Research

Cell line source(s)	For cAMP assays CHO-K1 (ATCC product CCL-61); E.coli (XL10) and BL21 (DE3) obtained from Agilent; Bg12 (P. pastoris) obtained from Biogrammatics
Authentication	All cell lines were authenticated by suppliers.
Mycoplasma contamination	CHO cells were determined to be free from mycoplasma as determined by a PCR-based assay, agar culture method, and Hoechst DNA stain method.
Commonly misidentified lines (See ICLAC register)	All cell lines were commercially available and are commonly used.
